# Comparative transcriptomic analysis of hematopoietic system between human and mouse by Microwell-seq

**DOI:** 10.1038/s41421-018-0038-x

**Published:** 2018-07-03

**Authors:** Shujing Lai, Wentao Huang, Yang Xu, Mengmeng Jiang, Haide Chen, Chen Cheng, Yingru Lu, He Huang, Guoji Guo, Xiaoping Han

**Affiliations:** 1 Center for Stem Cell and Regenerative Medicine, Zhejiang University School of Medicine, Hangzhou, Zhejiang 310058 China; 2 Stem Cell Institute, Zhejiang University, Hangzhou, Zhejiang 310058 China; 3 UT-ORNL Graduate School of Genome Science and Technology, University of Tennessee, Knoxville, TN 37996 USA; 4 College of Animal Science, Zhejiang University, Hangzhou, Zhejiang 310058 China; 5 Department of Critical Care Medicine, First Affiliated Hospital of Wenzhou Medical University, Wenzhou, Zhejiang 325000 China; 6 The 1st Affiliated Hospital, Zhejiang University School of Medicine, Hangzhou, Zhejiang 310003 China; 7 Institute of Hematology, Zhejiang University, Hangzhou, Zhejiang 310058 China; 8 Alliance for Atlas of Blood Cells, Tianjin, China

Dear Editor,

The classical model of hematopoiesis is a branched tree, rooted from long-term hematopoietic stem cell (LT-HSC) and followed by multipotent, oligopotent, and unipotent progenitor stages^1,2^. However, very limited studies have used systemic methods to investigate the heterogeneity of this population^3^. The cross-species comparison of hematopoietic hierarchy is also lacking. Here, through Microwell-seq, a high-throughput and low-cost scRNA-seq platform^4^ and a canonical correlation analysis computational strategy^5^, we conducted comparative transcriptomic analysis of hematopoietic hierarchy in human and mouse. We found that the hematopoietic stem and progenitor cell (HSPC) compartment in the two species is composed of subpopulations characterized by lineage-specific regulators, and the hematopoietic lineages and transcriptional profiling in hematopoiesis are well conserved among human and mouse, indicating an evolutionary similarity in their hematopoietic systems.

Using Microwell-seq^4^, we constructed a single-cell resolution transcriptomic atlas of HSPCs in human and mouse, having a total number of 71,729 single cells that are clustered into 39 subpopulations (44,914, 26,815 cells, and 20, 19 subpopulations were from human and mouse, respectively) by computational analysis (Fig. 1a, b). These subpopulations are further identified as undifferentiated HSPCs in a primed state or specific hematopoietic lineages by characteristic genes (Supplementary Fig. S1, Tables S1 and S2).

**Fig. 1:**
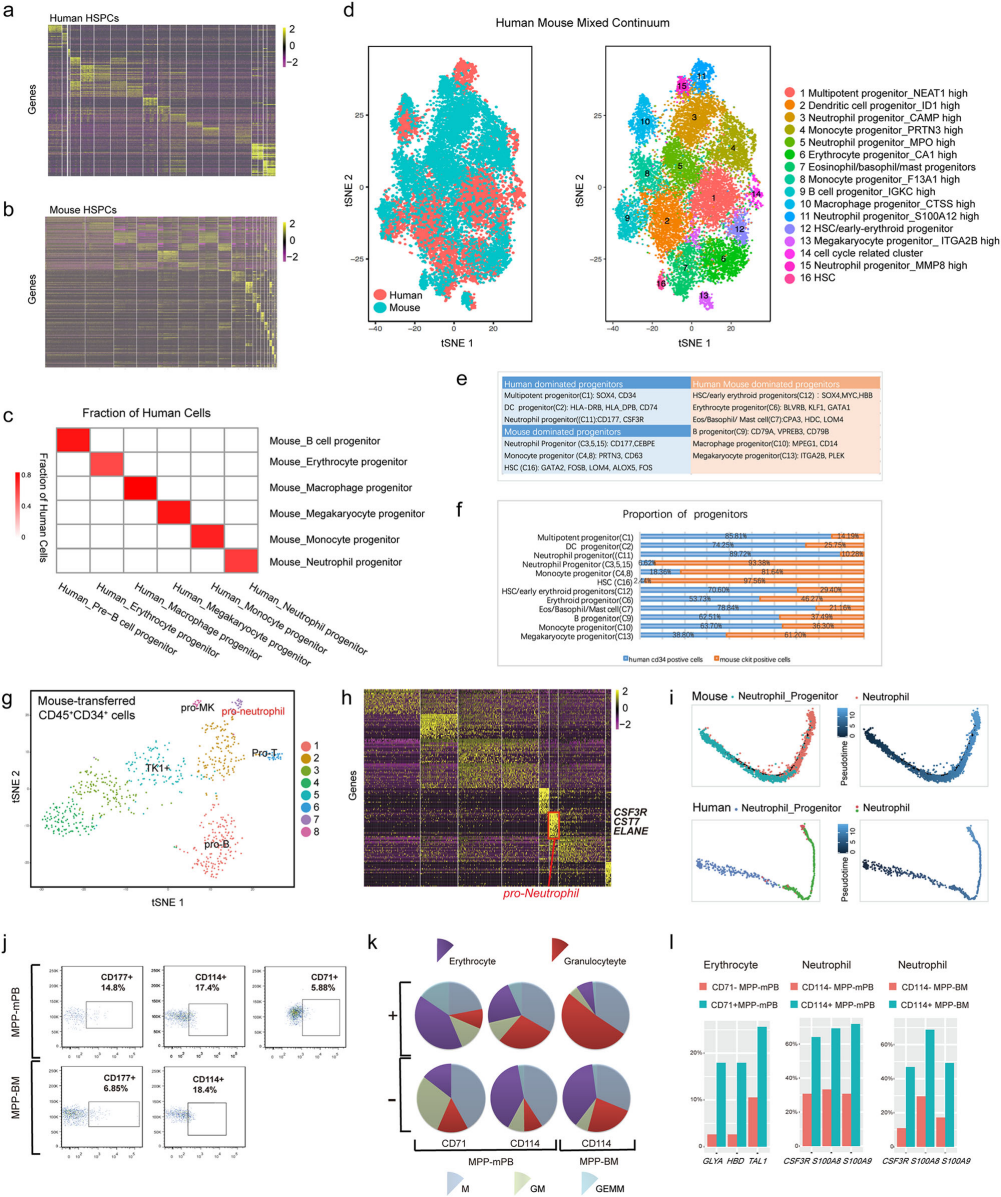
Comparative genomics analysis of hematopoietic system. **a** A gene expression heat map showing the differential gene expression for each cell cluster in human single hematopoietic stem and progenitor cell (HSPC) data. Rows correspond to individual genes found to be selectively upregulated in individual clusters; columns are individual cells, ordered by clusters. Yellow corresponds to high expression level; purple and black correspond to low expression level. **b** A gene expression heat map showing the differential gene expression for each cell cluster in mouse single HSPC data. The color scale and sample layout are the same as in Fig. 1a. **c** Fraction of cells in each human cluster was assigned to each mouse cluster based on orthologous genes. Red corresponds to high correlation level; white correspond to low correlation level. **d** t-Distributed Stochastic Neighbor Embedding (t-SNE) analysis of human-mouse HSPC compartment cell data. Cells are colored by experimental samples (left). Cells are colored by cell type cluster (right). **e** A table showing the confirmation and gene symbols of 16 cell types. **f** A bar showing the proportion of human and mouse cells of all progenitors. **g** t-SNE analysis of human CD45^+^CD34^+^ cells. Human peripheral blood CD34^+^ cells were transplanted into sublethally irradiated NCG mice, and human CD45^+^CD34^+^ cells were sorted from mouse bone marrow 2 months after transplantation. Cells are colored by cell type cluster. Lineage-primed progenitors like pro-neutrophil, pro-megakaryocyte, pro-T, and pro-B in CD45^+^CD34^+^ cells were visualized on the t-SNE map. **h** A gene expression heat map showing the differential gene expression for each cell cluster in human CD45^+^CD34^+^ cell data. Yellow corresponds to high expression level; purple and black correspond to low expression level. **i** The differentiation process of neutrophils and neutrophil progenitors from mouse and human datasets were plotted on the two-dimension space by Monocle. **j** The gating scheme of defined neutrophil progenitors (CD114^+^/CD177^+^) and erythrocyte progenitors (CD71^+^) from MPP (CD34^+^CD38^-^Thy1^-^CD45RA^-^CD49f^-^) cells. MPP cells were sorted from mobilized peripheral blood (mPB), bone marrow (BM) cells by FACS. **k** The percentage of different colony types in the colony-forming assay from CD71^+^ and CD71^−^ MPP-mPB, CD114^+^ and CD114^−^ MPP (mPB and BM). The results showed the average percentage from three independent experiments. **l** Single-cell qPCR was used to detect the expression of erythrocyte and neutrophil markers in the cells from colony-forming assay. The *y*-axes mean percentage of single cells expressing erythrocyte markers from CD71^+^ and CD71^−^ clones; or percentage of single cells expressing neutrophil markers from CD114^+^ and CD114^−^ clones

Furthermore, we explored the similarities and differences of HSPCs between two species. To obtain a detailed view on the cellular evolution from mouse to human in the HSPC system, we firstly switched mouse genes to human orthologous genes. Fraction of cells in each human cluster was assigned to each mouse cluster based on orthologous genes, and correlation level was shown in Fig. 1c. Cross-species correlation of orthologous genes shows gene expression conservation in the same cell type of different species. Moreover, further comparative transcriptomic analysis of hematopoietic system was performed. We used canonical correlation analysis (CCA) algorithm to perform human and mouse HSPC compartment integrated analysis^5^ (Fig. 1d). The CCA algorithm is a multivariate statistical technique for finding linear associations between two sets of variables that are maximally correlated. In scRNA-seq analysis, the CCA algorithm can detect the statistical common factors among two digital gene expression (DGE) matrices, which vary from each other due to batch effects or different methods used in normalization procedures.

Through computational CCA algorithm, we identified 16 subpopulations in human and mouse mixed HSPC continuum (Fig. 1d). Cluster 1 (C1), C2, C11, and C14 were dominated by human HSPCs, whereas C3–5, C8, and C15–C16 were occupied by mouse HSPCs (Fig. 1e). Six lineages (C6, C7, C9, C10, C12, C13) contain both human and mouse HSPCs (Fig. 1f). All clusters were distinguished by clearly reported differential gene expression modules^3,6–8^. Supplementary Table S3 showed detailed gene expression of all clusters, including known marker genes and novel transcription factors.

The human HSPC dominated clusters consisted of multipotent progenitor expressing *SOX4* and *CD34*, neutrophil progenitor expressing *CD177*, *CSF3R*, and *S100A9*, and dendritic cell progenitor showing high expression of *CD74* and MHCII-related genes (Supplementary Table S3)^6,9^. In mouse HSPC dominated lineages, we found three neutrophil progenitor clusters, two monocyte progenitor clusters and a hematopoietic stem cell cluster. The three neutrophil progenitors were characterized by high expression of *CAMP*, *LCN2*, *S100A8*, and *CEBPE*^3,6,8^. Both monocyte progenitor clusters showed high expression of monocyte marker *PRTN3*. One monocyte progenitor highly expressed *ELANE*, *MPO*, *CSTG*, and *CD63*, whereas the other one was defined by high expression of *F13A1*, *IRF8*, and *LY6E*^6^. Hematopoietic stem cell cluster is marked by high expression of *GATA2*, *FOS*, *LMO4*, *JUN*, and *ALOX5*.

Intriguingly, among the clusters having both human and mouse HSPCs, we identified erythroid progenitor, B-cell progenitor, macrophage progenitor, and megakaryocyte progenitor subpopulations, which express corresponding known lineage-specific markers, respectively. A cluster was thought to be eosinophil, basophil, and mast cell (eosin/baso/mast) common progenitors, with expression of eosin/baso/mast markers. We also found subpopulations showing combined expression of hematopoietic stem cell and erythroid genes (HSC/early-ERY). Meanwhile, the human and mouse cells within the same clusters still showed differential expression of orthologous gene modules (Supplementary Fig. S2). For example, *SOX4* and *MYC* were expressed 100-fold higher in human-derived HSC/early erythroid progenitor cells, and *CDK4* was expressed higher in mouse-derived HSC/early erythroid progenitor cells.

To test the stability of the hematopoietic differentiation model in vivo, we performed xenotransplantation experiment to validate the hematopoietic hierarchy model revealed by our computational analysis. We transplanted 1 × 10^5^ of mobilized peripheral blood (mPB) CD34^+^ cells into sublethally irradiated NCG mouse via femur cavity. Two months after transplantation, human CD45^+^CD34^+^ cells in mouse femur and tibia were sorted by FACS. We found that the multipotent to unipotent hierarchy in the human hematopoietic system is largely revealed in the xenograft experiment (Fig. 1g, h). Through GO analysis in scRNA-seq data of these human CD45^+^CD34^+^ cells, we found that human HSPCs changed its pathways after the xenograft experiment (Supplementary Fig. S3). Erythroid-primed progenitor stimulated metabolism-related pathways like carbon metabolism. Human HSPCs reside in specific niches that provide various instructive cues regulating their self-renewal and development. Suda et al.^10^ proposed that niche can improve metabolic regulation of hematopoietic system. Erythrocyte cell is the fastest self-renewing cell type (the hematopoietic system generates 2 × 10^11^ erythrocytes per day), the distinct metabolism pathways may affect the self-renewal capacity of erythrocytes.

Our study also resolves the maturation trajectory from neutrophil progenitor to mature neutrophil lineages among human and mouse (Fig. 1i). Pseudotime from other unipotent progenitor to mature lineages was established to show unipotent progenitor exactly earlier than mature lineages (Supplementary Fig. S4).

To further validate the lineage-primed progenitors and their early differentiation pattern, we searched for cell surface markers that can characterize different clusters. We used CD41, TFRC (CD71), CSF3R (CD114), CSF1R (CD115), and MHCII to detect early megakaryocytic, erythroid, neutrophil, monocyte, and dendritic progenitors, respectively. With specific markers, we could separate at least five kinds of lineage-biased progenitors from human MPP (CD34^+^CD38^-^Thy1^-^CD45RA^-^CD49f^-^) cells, HSC (D34^+^CD38^−^Thy1^+^CD45RA^-^CD49f^+^) and CMP (CD34^+^CD38^+^CD45RA^-^) cells, respectively (Supplementary Fig. S5).

The phylogenetic tree indicated that neutrophil progenitors were highly conserved between human and mouse (Supplementary Fig. S6). Consequently, we chose neutrophil as a candidate and used colony-forming assays and single-cell quantitative PCR (qPCR) to validate our computational observation. We also chose erythrocyte as a candidate for its good performance in colony-forming assay. The gating scheme defined neutrophil progenitors (CD114^+^/CD177^+^) and erythrocyte progenitors (CD71^+^) from MPP cells sorted from peripheral blood cells and bone marrow (BM) cells (Fig. 1j). Single-cell colony-forming assay showed that CD71^+^ selection remarkably enriched CFU-E generating cells. Forty-one percent of clones generated by CD71^+^ MPP-mPB were CFU-E, threefold higher than that in CD71^−^ MPP-mPB. Meanwhile, CD114^+^ selection enriched CFU-G generating cells. The percentage of CFU-G generating cells in CD114^+^ MPP-mPB is three times as large as that in CD114^−^ MPP-mPB. We found similar clone results in MPP-BM (Fig. 1k). Single-cell qPCR assay suggests that CD71 and CD114 are able to enrich erythroid-primed progenitor and neutrophil-primed progenitor from MPP stage, respectively (Fig. 1l, Supplementary Table S4 and Fig. S7).

In conclusion, our comparative transcriptomic analysis of hematopoietic system revealed an evolutionary conservation in the hematopoietic hierarchy across human and mouse. The xenotransplantation experiments in immunodeficient mice contributed to our understanding that niche might play a role in the species-specific cellular phenotypes. The Microwell-seq platform and comparative single-cell transcriptome analysis method should be widely applicable to other systems.

## Data access

Raw sequencing data and DGE data are accessible through the Gene Expression Omnibus (GEO) accession code GSE92274.

## Electronic supplementary material

Supplementary methods and figures

Table S1

Table S2

Table S3

Table S4
