# Supplementary material for: Comparative transcriptomic analysis of hematopoietic system between human and mouse by Microwell-seq
Source: Cell Discov. 2018 Jul 3;4:34. doi: 10.1038/s41421-018-0038-x (PMC6028447; doi:10.1038/s41421-018-0038-x)
Supplement: Supplementary file 1 — Supplementary methods and figures [file 41421_2018_38_MOESM1_ESM.pdf]

Supplementary methods and Figures

Including Materials and Methods, 7 figures.

Materials and Methods

Human CD34$^+$ mobilized peripheral blood collection and process

Human mobilized peripheral blood samples were collected from donors at The First Affiliated Hospital of Zhejiang University School of Medicine (Zhejiang, China). The donors have signed the informed consent form. All procedures are approved by the Ethical Committee on Medical Research at School of Medicine. mPB CD34$^+$ cells were selected by human CD34 selection kit (StemCell Technologies, Cat# 18056), following the protocol provided by the manufacturer. After spinning at 300× *g* for 5min, the supernatant was removed and mPB CD34$^+$ cells were suspended in 1× DPBS with 2 mM EDTA. For Microwell-seq, cells were diluted to ~100,000/mL.

Mouse cKit$^+$ hematopoietic cells collection and process

Here we used high-throughput single-cell RNA sequencing combined with fluorescence-activated cell sorting of mouse cKit$^+$ hematopoietic cells. Firstly, mouse bone marrow cells were suspended in 100 μL of DPBS with 5% FBS for exposure with cKit antibodies (eBioscience, Cat# 12598182). Then, we sorted HSCs and progenitor cells by a BD FACSAria II cell sorter. Cells were diluted in DPBS with 2 mM EDTA before Microwell-seq experiments.

Immunodeficient mouse transplantation

mPB CD34$^+$ cells were transplanted into sublethally irradiated NCG (NOD-Prkdc^{em26Cd52}Il2rg^{em26Cd22}/Nju, Nanjing Biomedical Research Institute of Nanjing University) mice via an injection into the femur cavity (~1 × 10^5 cells per mouse). Two months after transplantation, human CD45$^+$CD34$^+$ cells were sorted from mice bone marrow cells collected from the femur and tibia. NCG mice were fed in Laboratory Animal Center of Zhejiang University and all procedures were approved by the Ethical Committee on Laboratory Animal Center of Zhejiang University.

Cell sorting by FACS

Human CD34$^+$ cells were suspended in 100 μL of DPBS with 5% FBS for staining of antibodies. The following antibodies were used for cell sorting: CD34 PE (Biolegend, clone 581, Cat #343506), CD34 FITC (BD bioscience, clone 581, Cat# 555821), CD38 PE-Cy7 (BD bioscience, clone HIT2, Cat# 560677), CD45RA APC/Fire 750 (Biolegend, clone HI100, Cat# 304151), CD49f BV421 (Biolegend, clone GoH3, Cat# 313623), CD74 PE (Biolegend, clone LN2, Cat# 326807), CD90 APC (BD bioscience, clone 5E10, Cat# 559869), CD114 PE (Biolegend, clone LMM741, Cat# 346105), CD115 PE (Biolegend, clone 9-4D2-1E4, Cat# 347303), CD71 PE (BD bioscience, clone M-A712, Cat# 561938), CD41 FITC (BD bioscience, clone HIP8, Cat# 561851), MHCii FITC (BD bioscience, clone Tu39, Cat# 562008). Cell sorting was performed on the BD FACSAria II.

Colony forming assays

Colony forming assays for each population were performed by plating 1000 FACS sorted cells into into 6-well plate with 3 ml MethoCult™ H4034 Optimum methylcellulose-based medium (StemCell Technologies). Three independent experiments were performed for each population. After 14 days in culture, CFU-multilineage colonies were calculated according to instructions from StemCell Technologies.

Single-cell qPCR

39 cells from CD71$^+$ clones, 38 cells from CD71$^-$ clones, 39 cells from CD114$^+$ clones and 39 cells from CD114$^-$ clones were randomly picked from each clone. Individual primer sets (total of 96) were pooled to a final concentration of 0.1 μM for each primer. Individual cells picked directly into 8-well strips loaded with 5 μL RT-PCR master mix (2.5 μL CellsDirect reaction mix, Invitrogen; 0.1 μL RT/Taq enzyme, Invitrogen; 0.5 μL primer pool; nuclease free water to 20 μL) in each well. Strips were immediately frozen on dry ice. After brief centrifugation at 4 °C, Strips were placed immediately on PCR machine. Cell lyses and sequence-specific reverse transcription were performed at 50 °C for 60 min. Then, reverse transcriptase inactivation and Taq polymerase activation was achieved by heating to 95 °C for 3 min. Subsequently, in the same tube, cDNA was subjected to 20 cycles of sequence-specific amplification by denaturing at 95 °C for 15 s, annealing and elongation at 60 °C for 15 min. Pre-amplified products were diluted 5-fold prior to analysis. Amplified single cell samples

were analyzed with Universal PCR Master Mix (Applied Biosystems), EvaGreen Binding Dye (Biotium) and individual qPCR primers using 96 × 96 Dynamic Arrays on a BioMark System (Fluidigm). Ct values were calculated using the BioMark Real-Time PCR Analysis software (Fluidigm).

Preprocessing of Microwell-seq Data

Drop-seq core computational tool was used for the preprocessing of Microwell-seq data. The quantification and statistical analysis was described in the study of Han, X. et al[4]. Cellular barcode and unique molecular identifier (UMI) were extracted from Read One, we discarded the paired reads if the quality of any base in the barcode was below 10, and only Read Two was used for alignment. We used STAR (version 2.5.1b) with default parameters for mapping.

Reads from human mPB $CD34^+$ cells, human mPB $CD34^-$ cells and mouse-transferred $CD34^+$ cells were aligned to GRCh38 human genome. Reads from mouse $cKit^+$ cells, mouse bone marrow cells were aligned to mouse genome. All multi-aligned reads were removed and GTF annotation files from Gencode were used to tag aligned reads. For UMI count, molecular barcodes with one edit distance were merged to one within a gene. For the cell quality control, we excluded cells where less than 500 genes were expressed. High proportion (> 10%) of transcript counts derived from mitochondria encoded genes may indicate low cell quality and we removed these unqualified cells from downstream analysis. Eventually, 95,569 cells were preserved: 914 mouse-transferred $CD34^+$ cells; 44,914 human mPB $CD34^+$ cells from 7 batches

(7353, 13690, 2375, 9583, 2741, 1368 and 7804 cells respectively; and 4,973 mPB $CD34^-$ cells. 26815 mouse $cKit^+$ cells, 14,853 mouse bone marrow cells.

We used canonical correlation analysis (CCA) algorithm to perform comparative transcriptomic analysis of hematopoietic system between human and mouse. The CCA algorithm is a multivariate statistical technique for finding linear associations between two sets of variables that are maximally correlated. In scRNA-seq analysis, the CCA algorithm can detect the statistical common factors amongst two digital gene expression (DGE) matrices which vary from each other due to batch effects or different methods used in normalization procedures. These factors are aggregations of conserved gene-to-gene correlations between human and mouse, and therefore we can align all human cells against all mouse cells in an identical linear space and visualize shared subpopulations in different species with further analysis like t-SNE.

Supplementary Figure 1

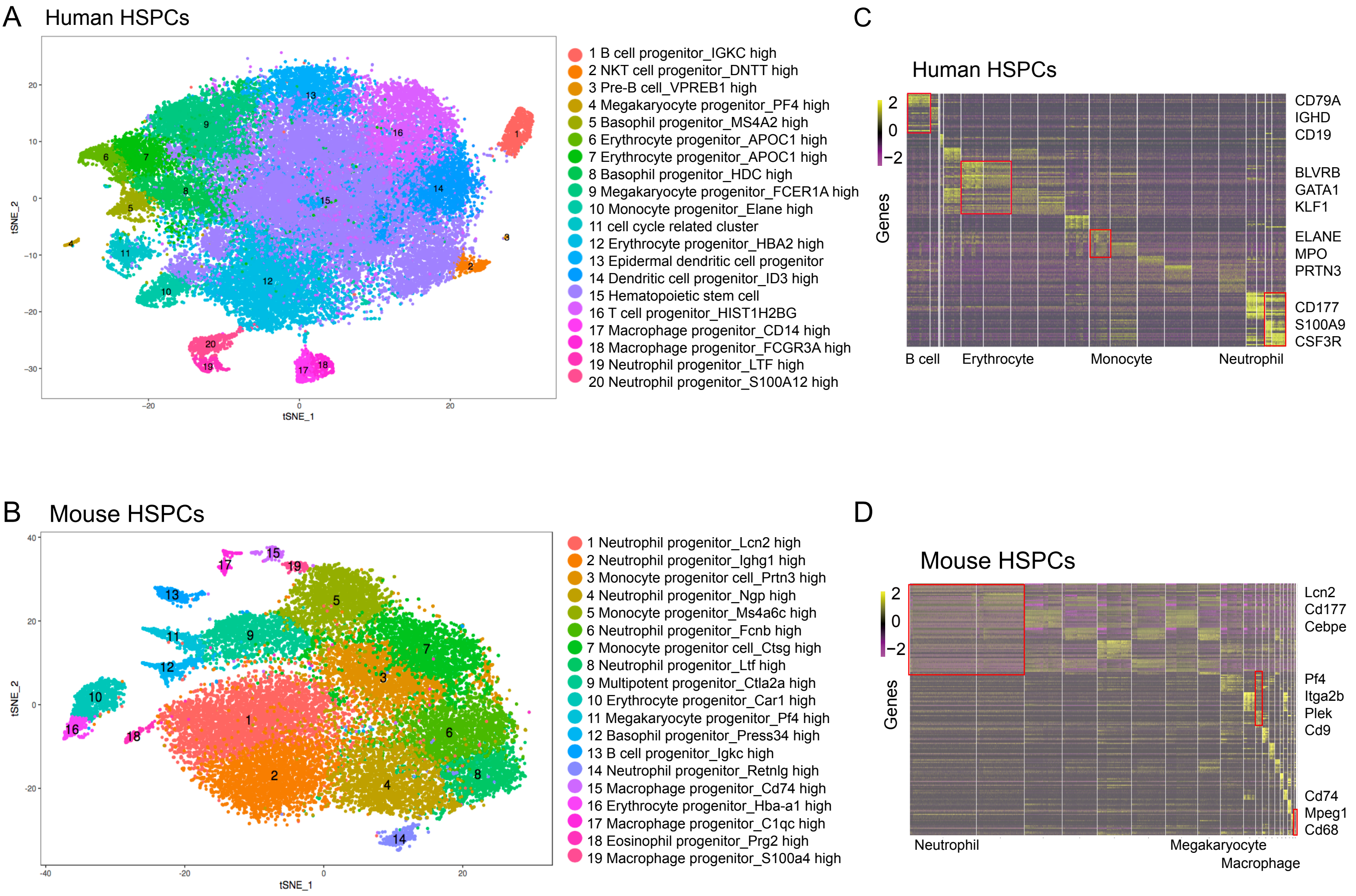

Supplementary Figure S1 Resolving Cellular Heterogeneity in the human and mouse hematopoietic stem and progenitor cells. (A) A t-Distributed Stochastic Neighbor Embedding (t-SNE) map of human single hematopoietic stem and progenitor cell (HSPC) data. Cells are colored by cell type cluster. (B) A t-SNE map of mouse HSPC data. Cells are colored by cell-type cluster. (C) A gene expression heat map showing the top differentially expressed genes for each cell cluster in human HSPC data. Yellow corresponds to high expression level; purple and black correspond to low expression level. (D) A gene expression heat map showing the top differentially expressed genes for each cell cluster in mouse HSPC data. Yellow corresponds to high expression level; purple and black correspond to low expression level.

Supplementary Figure 2

A

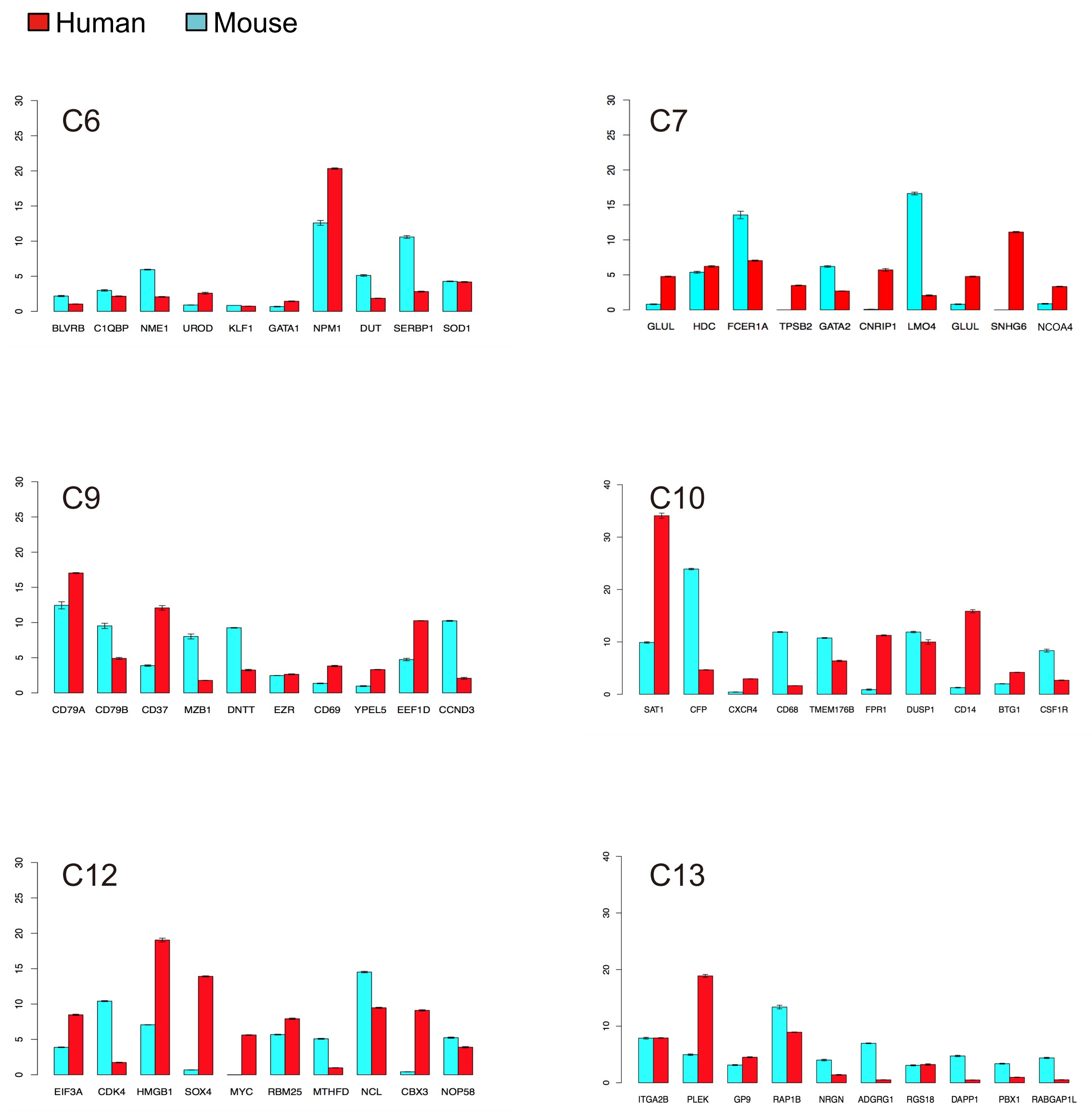

B

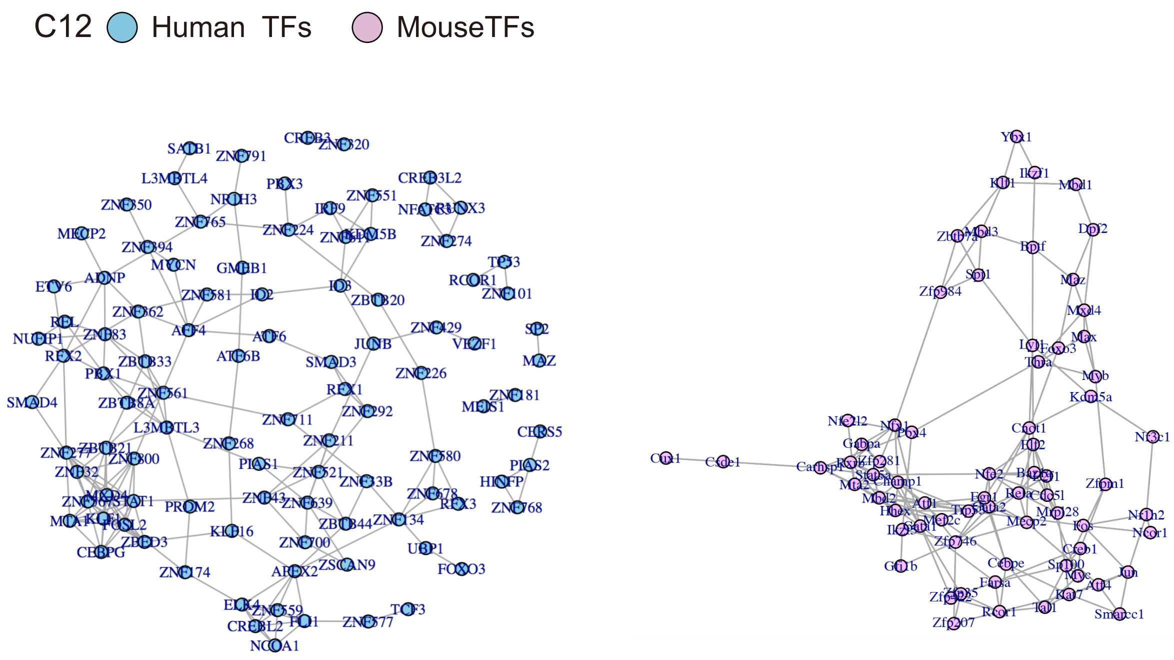

Supplementary Figure S2 Comparative gene expression difference among human and mouse overlap progenitors through CCA analysis. (A) We chose top genes of six human and mouse dominated progenitors mentioned in Fig. 1e, f (C6, C7, C9, C10, C12, C13). The bar chart describes the distinct of gene expression among six subpopulations. (B) We chose the HSC/early-erythroid progenitor (C12) as a candidate, to compare the similarity and differences of TFs pathway between human and mouse.

Supplementary Figure 3

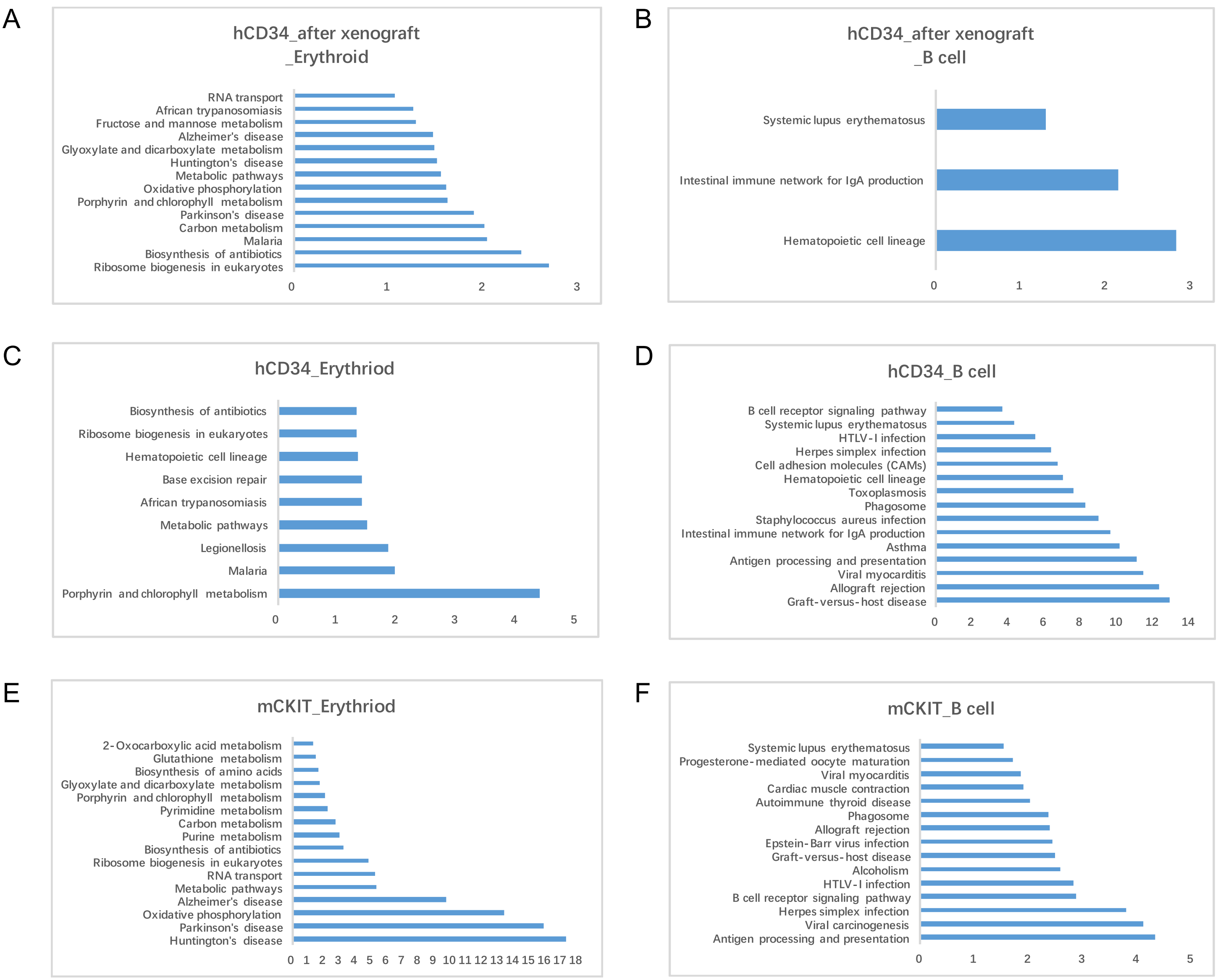

Supplementary Figure S3 Pathway of erythroid and B cell progenitors with different niches. Go analysis of erythroid and B cell progenitors from different samples: (A, B) human $CD45^{+}CD34^{+}$ cells sorted from mouse bone marrow two months after transplantation, (C, D) human single hematopoietic stem and progenitor cells, (E, F) mouse single hematopoietic stem and progenitor cells. The Go terms were selected to show with $P < 0.05$. *P* value was shown as -log10 (*P* value).

Supplementary Figure 4

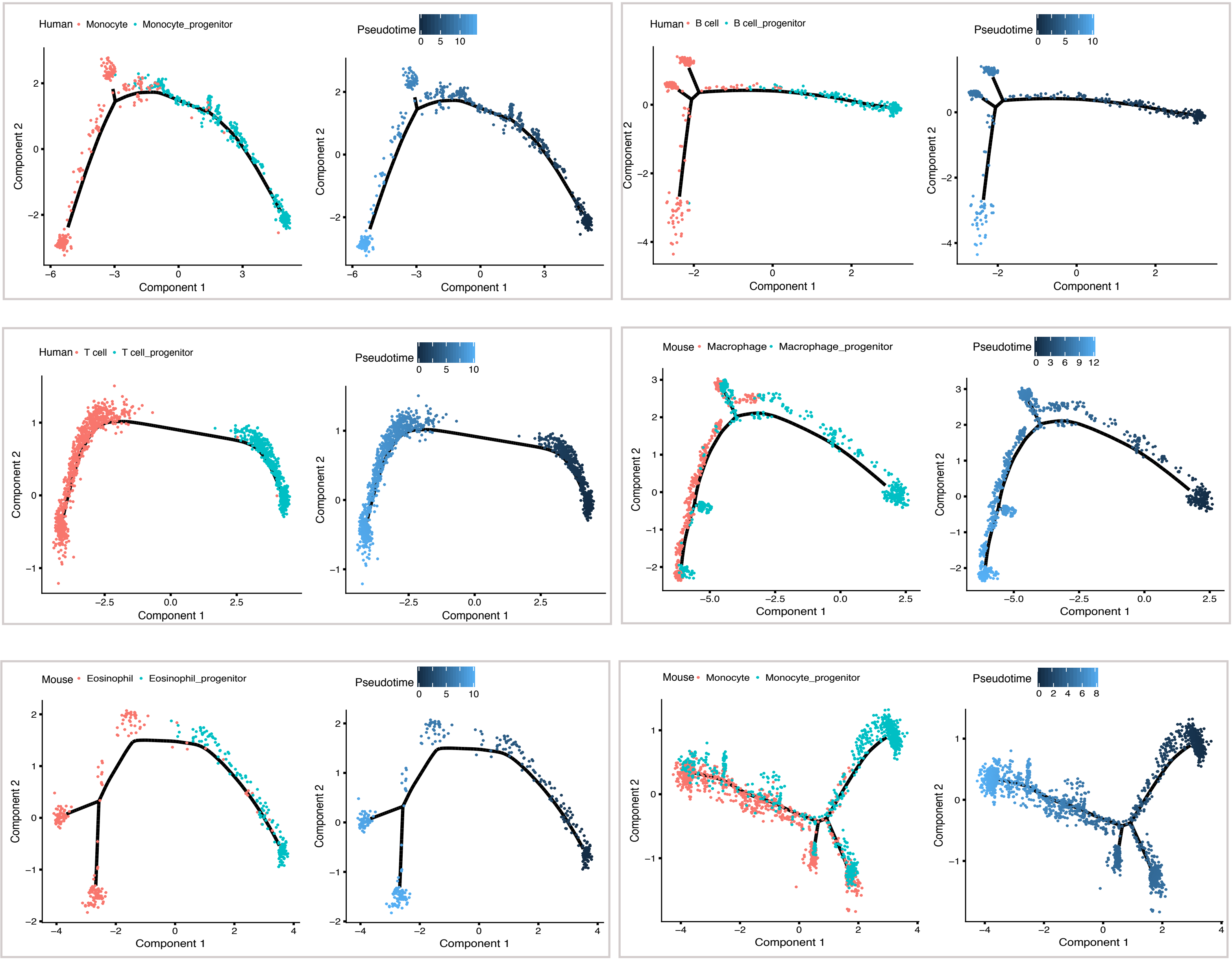

Supplementary Figure S4 The pseudotime of unipotent progenitors to mature lineages. The maturation trajectory from unipotent progenitors to mature lineages are plotted on the 2-dimension t-SNE subspace reduced by Monocle.

Supplementary Figure 5

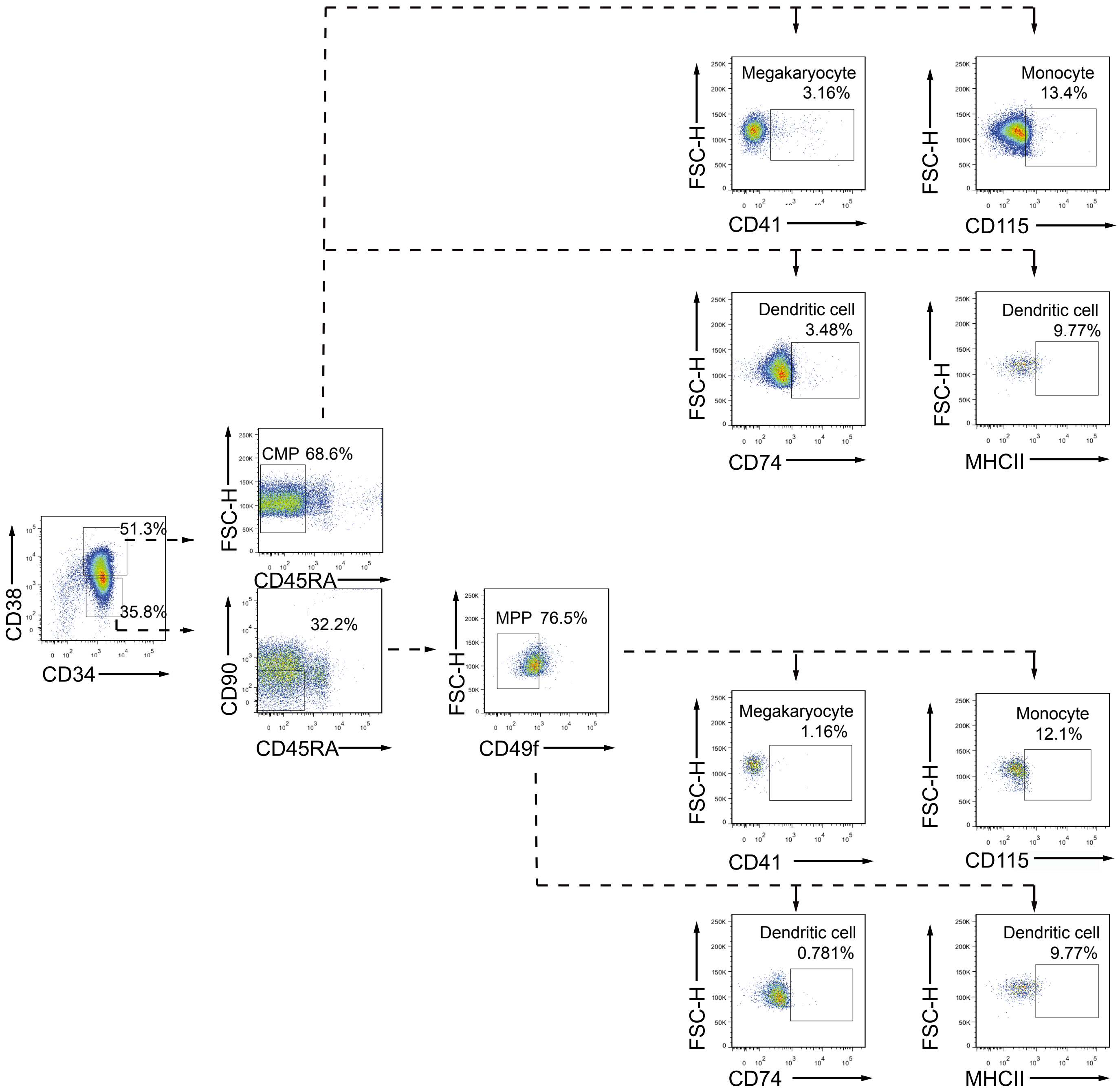

Supplementary Figure S5 Confirmed unipotent progenitors by FACS. The gating scheme of defined unipotent progenitors from MPP ($CD34^+CD38^-Thy1^-CD45RA^-CD49f^-$) and CMP ($CD34^+CD38^+CD45RA^-$). Monocyte progenitors ($CD34^+CD38^-Thy1^-CD45RA^-CD49f^-CD115^+$ or $CD34^+CD38^+CD45RA^-CD115^+$), dendritic cell progenitors ($CD34^+CD38^-Thy1^-CD45RA^-CD49f^-CD74^+$/MHCII or $CD34^+CD38^+CD45RA^-$ $CD74^+$/MHCII) and megakaryocyte progenitors ($CD34^+CD38^-Thy1^-CD45RA^-CD49f^-CD41^+$ or $CD34^+CD38^+CD45RA^-CD41^+$) were confirmed in MPP and CMP.

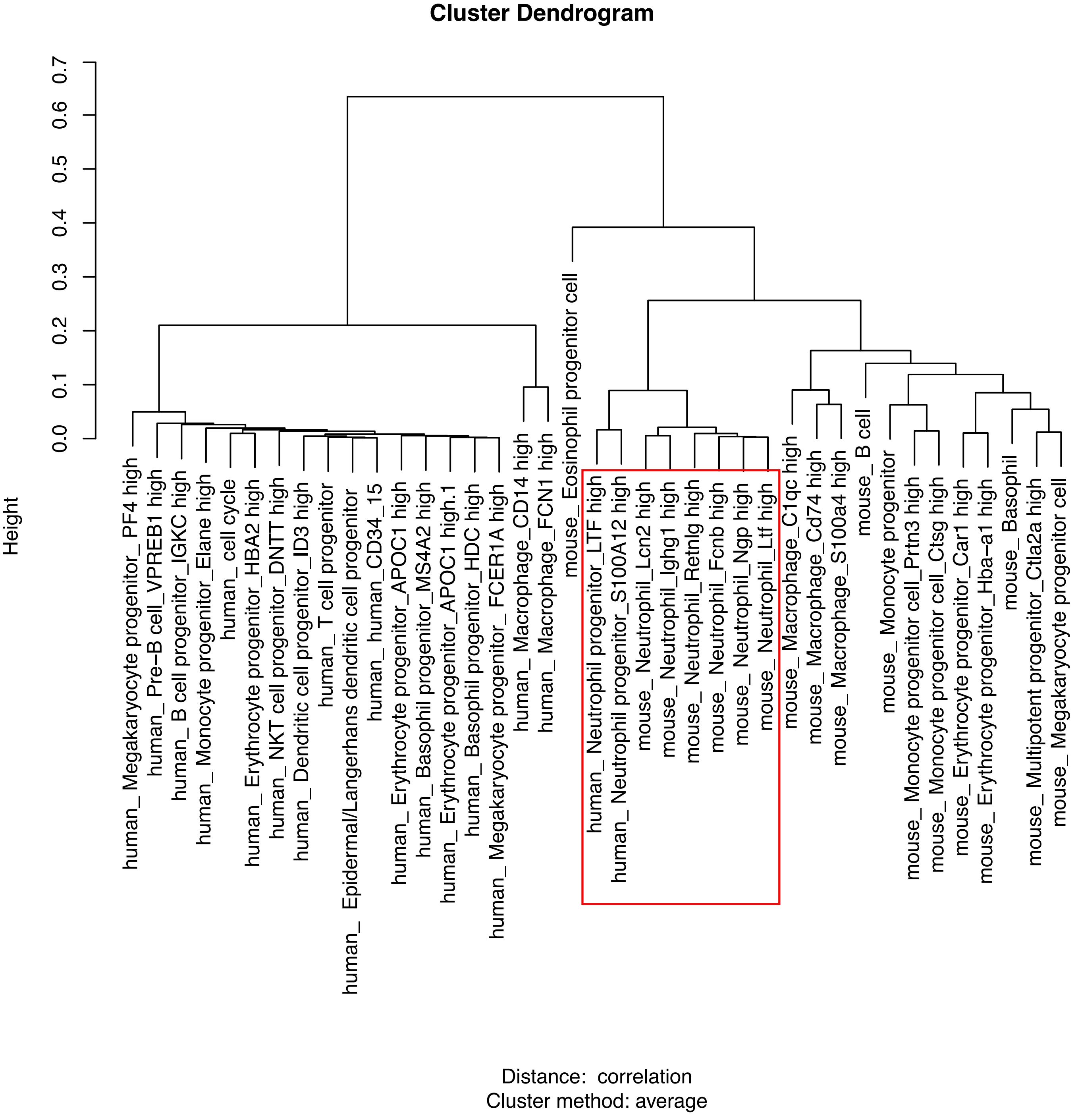
Cluster Dendrogram
Height
0.0
0.1
0.2
0.3
0.4
0.5
0.6
0.7
human_ Megakaryocyte progenitor_ PF4 high
human_ Pre−B cell_VPREB1 high
human_ B cell progenitor_IGKC high
human_ Monocyte progenitor_Elane high
human_ cell cycle
human_ Erythrocyte progenitor_HBA2 high
human_ NKT cell progenitor_DNTT high
human_ Dendritic cell progenitor_ID3 high
human_ T cell progenitor
human_ Epidermal/Langerhans dendritic cell progenitor
human_ human_CD34_15
human_ Erythrocyte progenitor_APOC1 high
human_ Basophil progenitor_MS4A2 high
human_ Erythrocyte progenitor_APOC1 high.1
human_ Basophil progenitor_HDC high
human_ Megakaryocyte progenitor_ FCER1A high
human_ Macrophage_CD14 high
human_ Macrophage_FCN1 high
mouse_ Eosinophil progenitor cell
human_ Neutrophil progenitor_LTF high
human_ Neutrophil progenitor_S100A12 high
mouse_ Neutrophil_Lcn2 high
mouse_ Neutrophil_Ighg1 high
mouse_ Neutrophil_Retnlg high
mouse_ Neutrophil_Fcnb high
mouse_ Neutrophil_Ngp high
mouse_ Neutrophil_Ltf high
mouse_ Macrophage_C1qc high
mouse_ Macrophage_Cd74 high
mouse_ Macrophage_S100a4 high
mouse_ B cell
mouse_ Monocyte progenitor
mouse_ Monocyte progenitor cell_Prtn3 high
mouse_ Monocyte progenitor cell_Ctsg high
mouse_ Erythrocyte progenitor_Car1 high
mouse_ Erythrocyte progenitor_Hba−a1 high
mouse_ Basophil
mouse_ Multipotent progenitor_Ctla2a high
mouse_ Megakaryocyte progenitor cell
Distance: correlation
Cluster method: average

Supplementary Figure S6 The phylogenetic tree of 39 clusters. Average expression across 9,242 homologous genes of human and mouse was calculated for 19 cell clusters of mouse and 20 cell clusters of human, and the relative distance between homologous-gene-expression patterns for all clusters are represented by a phylogenetic tree.

Supplementary Figure 7

A

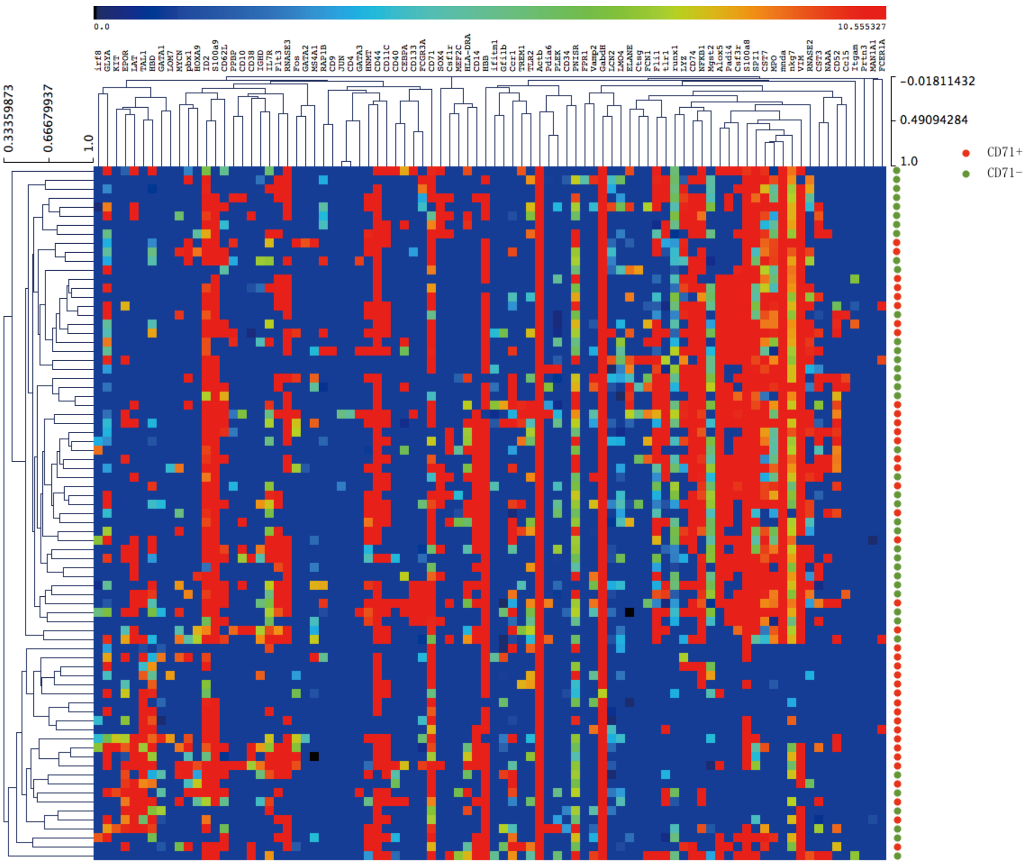

B

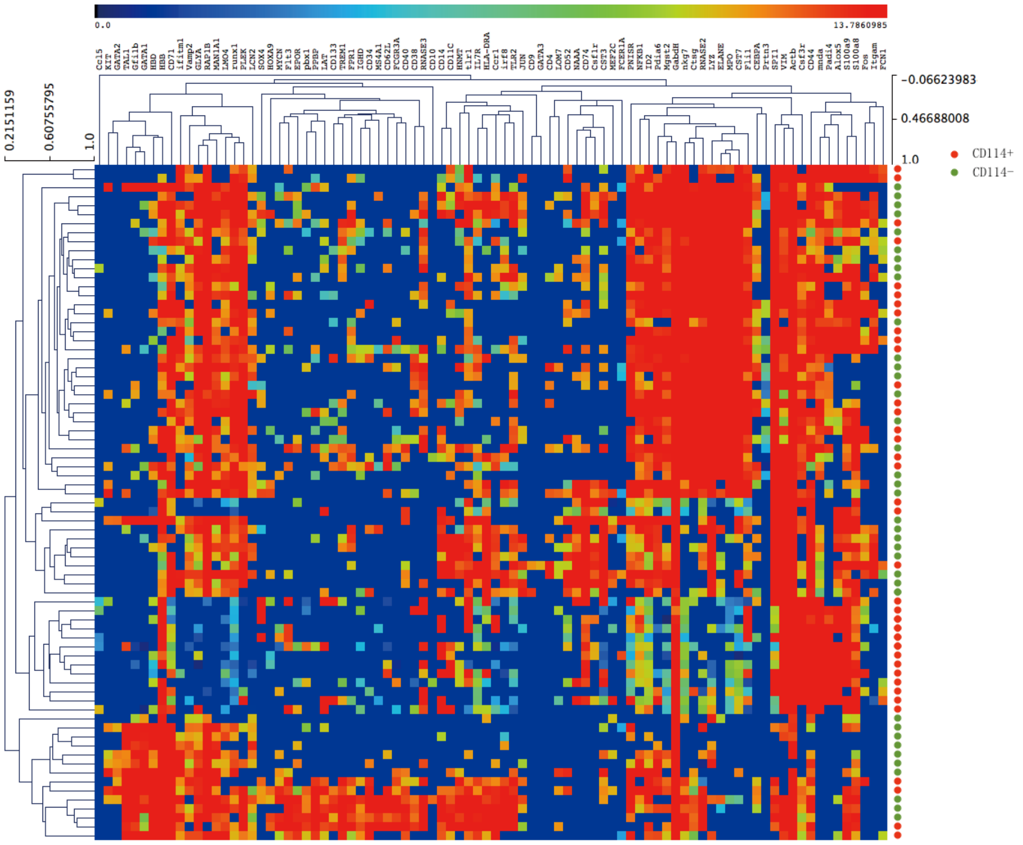

Supplementary Figure S7 Single cell qPCR results from colony forming assays. (A) A heat map showing that the unbiased hierarchical clustering well separates single-cell gene expression signatures from 39 cells from $CD71^+$ MPP-mPB clones and 38 cells from $CD71^-$ MPP-mPB clones. Each row corresponds to a specific gene; each column corresponds to a particular single cell. Red to yellow suggest high to middle expression, whereas green to blue suggest low to no expression. (B) A heat map showing that the unbiased hierarchical clustering well separates single-cell gene expression signatures from 39 cells from $CD114^+$ MPP-mPB clones and 39 cells from $CD114^-$ MPP-mPB clones. Columns correspond to individual genes; rows are individual cells. Red corresponds to high expression level; blue and black correspond to low expression level.
